# Preparation of Cement Clinker from Geopolymer-Based Wastes

**DOI:** 10.3390/ma14216534

**Published:** 2021-10-30

**Authors:** Rabii Hattaf, Mohamed Benchikhi, Abdessamad Azzouzi, Rachida El Ouatib, Moussa Gomina, Azzeddine Samdi, Redouane Moussa

**Affiliations:** 1 Laboratory of Physics and Chemistry of Inorganic Materials, Faculty of Sciences Aïn Chock, University Hassan II Casablanca, Casablanca 53306, Morocco; rab.hattaf@gmail.com (R.H.); azzouzi.abdessamad28@gmail.com (A.A.); elouatib@yahoo.fr (R.E.O.); azdn.samdi@gmail.com (A.S.); redmoussa@yahoo.fr (R.M.); 2 Department of Chemistry, Faculté Polydisciplinaire, University Sultan Moulay, Slimane, Mghila, BP.592, Beni-Mellal 23000, Morocco; 3 CRISMAT UMR6508 CNRS, ENSICAEN, 6 Boulevard Maréchal Juin, CEDEX 4, 14050 Caen, France; moussa.gomina@ensicaen.fr

**Keywords:** geopolymer, waste, clinker, alternative raw materials

## Abstract

In order to avoid potential environmental pollution from geopolymer-based material wastes, this work investigated the feasibility of using these materials as alternative raw materials in the preparation of cement clinker. The geopolymer binders and mortars were used as substitutes for natural mineral clays since they are rich in silica and alumina. Simulated geopolymer wastes were prepared by the activation of metakaolin or fly ash by an alkaline silicate solution. The cement-clinkers fired at 1450 °C for 1h were characterized by XRD, XRF, SEM-EDS, and a free lime (CaO_f_) content test. The anhydrous clinker mineral phases C_3_S (Ca_3_SiO_5_), C_2_S (Ca_2_SiO_4_), C_3_A (Ca_3_Al_2_O_6_), and C_4_AF (Ca_4_Al_2_Fe_2_O_10_) were well-crystallized in all investigated formulations. The free lime was lower than 1.3 wt% in all elaborated clinkers, which indicates a high degree of clinkerization. The results demonstrate that geopolymer binder and mortar materials are suitable substitutes for natural mineral clay incement clinker preparation.

## 1. Introduction

Geopolymers are a class of alkaline aluminosilicate materials, synthesized by the activation of various reactive aluminosilicate materials of geological origin (e.g., metakaolin) or industrial by-products (e.g., fly ash) by a concentrated alkali hydroxide silicate solution. These materials have several advantages: easy preparation, low curing temperature (i.e., ≤100 °C), reduced gas emissions, and good properties [1,2]. For these reasons, geopolymer materials are considered a promising and sustainable ecological alternative to conventional materials, especially cement and ceramics.

In recent years, geopolymer materials have been adopted in many fields, including construction and civil engineering (as binders, mortars, concretes, light weight panels and bricks for thermal and acoustic insulation, protective coating, low-cost ceramics, and fire protection structures) [2,3,4,5,6,7]. These fields are considered among the highest producers ofsolid waste in the world. Therefore, it is essential to propose anticipatory strategies in parallel for the good management and elimination of this type of waste. These strategies must accompany the progressive introduction of these materials into the construction materials market, and consequently avoid their accumulation in huge quantities or their disposal in landfills. In addition, environmental concerns have become a global priority in recent decades, pushing towards sustainable practices for the recovery of waste and by-products. Furthermore, the valorization of geopolymer-based material wastes can contribute to the conservation of finite, natural, non-renewable resources. In this context, a very limited number of initiatives have focused on the recycling of geopolymer waste. These studies generally remain within the framework of a single approach, which consists in reusing the waste in the form of aggregates to replace natural aggregates (sand or gravel) in order to produce mortars or concretes. Hattaf et al. [8] proposed to reuse geopolymer waste as a substitute for the starting raw materials (metakaolin and fly ash) to produce new geopolymer matrices. These authors demonstrated that high compressive strengths can be maintained for substitution rates up to 40 wt% (compressive strengths greater than 43 MPa). A. Akbarnezhad and S. Mesgari [9] studied the substitution of natural aggregates by recycled geopolymer concrete wastes in the form of coarse aggregates for the production of geopolymer or Portland cement concretes. Their results showed that the total substitution of natural aggregates resulted in only 12.9, 10.7, and 15.2% decreases in compressive strength, modulus of elasticity, and modulus of rupture, respectively. P. Zhu et al. [10] studied the replacement of river sand aggregates by recycled geopolymer aggregates for the preparation of metakaolin-based mortars. They showed that the mechanical strength of the prepared mortars is unchanged up to a substitution rate of about 50 wt%. A. Gharzouni et al. [11] developed metakaolin-based geopolymer matrices by substituting metakaolin with relatively fine aggregates of recycled geopolymers. The results showed that the feasibility of these materials is limited to a substitution rate of about 20%.

Geopolymer binders and mortars are very rich in silica and alumina. Thus, these materials have considerable potential as substitutes for natural mineral clay in the preparation of cement clinkers. To the best of our knowledge, the valorization of geopolymer materials in clinker production has not yet been reported. Therefore, this work aims to prove the feasibility of using geopolymer binders and mortar powders as cement raw materials. The cementclinkers were prepared by partially replacing natural mineral clay with geopolymer binders or mortar powders. The simulated geopolymer materials were obtained throughthe activation of metakaolin or fly ash by an alkaline silicate solution. The prepared materials were characterized mainly by X-ray diffraction (XRD), scanning electron microscopy coupled with energy-dispersive X-ray spectroscopy (SEM-EDS), and X-ray fluorescence (XRF).

## 2. Materials and Methods

### 2.1. Materials, Equipment, and Methods

The metakaolin (MK) used in this work was supplied by Imerys company reference Agrical M1000 (Paris, France). The fly ash (FA) was obtained from the JarfLasfar thermal power station (El Jadida, Morocco). The silica fume (SF) used in this work was exploited by a local cement manufacturing company. The standard sand used to prepare the mortars was provided by Nouvelle du Littoral Company (Leucate, France). The sodium silicate solution (45 wt%) with molar ratio SiO_2_/Na_2_O = 2 was supplied by Cadilhac Society. Sodium hydroxide NaOH (98%), calcium carbonate CaCO_3_ (99%), and iron oxide Fe_2_O_3_ (99%) used in this work were of analytical grade.

The chemical composition of powders was determined via wavelength dispersive X-ray fluorescence analysis (WDXRF). The WDXRF measurements were performed using a Thermo ARL 9800XP spectrometer (Thermofisher^®^, Waltham, MA, USA)equipped with an X-ray tube, with a maximum power of 3.0 kW. (30 kV and 80 mA). It was calibrated using certified reference material standards (CRMs) from the same matrix as the samples to be analyzed. A calibration curve was established for each element, and the correlation coefficients were determined by regression calculation. The content of the elements was evaluated by using the ADVANTX-2252 instrument operating with v2.6.3.3985D of the OXSAS software (Thermofisher^®^, Waltham, MA, USA). The samples were prepared by forming them into fused glass discs. To prepare the glass disc samples, 4 g of the dried sample was mixed with 4 g of dilithium tetraborate (spectroflux 100) andwas then melted in a furnace at 1000 °C for 1h.After that, the homogeneous melted sample was recast into glass beadsthat were 2 mm thick and 32 mm in diameter. The loss on ignition (LOI) was measured at 1000 °C. The chemical composition of metakaolin, fly ash, limestone, silica fume, and sand was determined by using X-ray fluorescence;this issummarized in Table 1. The metakaolin and fly ash are mainly composed of silica and alumina. It should be noted that the fly ash contains an appreciable amountofFe_2_O_3_, CaO, and MgO. Sand and silica fume powders are mainly composed of SiO_2_. The limestone powder contains more than 56% of CaO, with an ignition loss of 43%.

**Figure 1:**
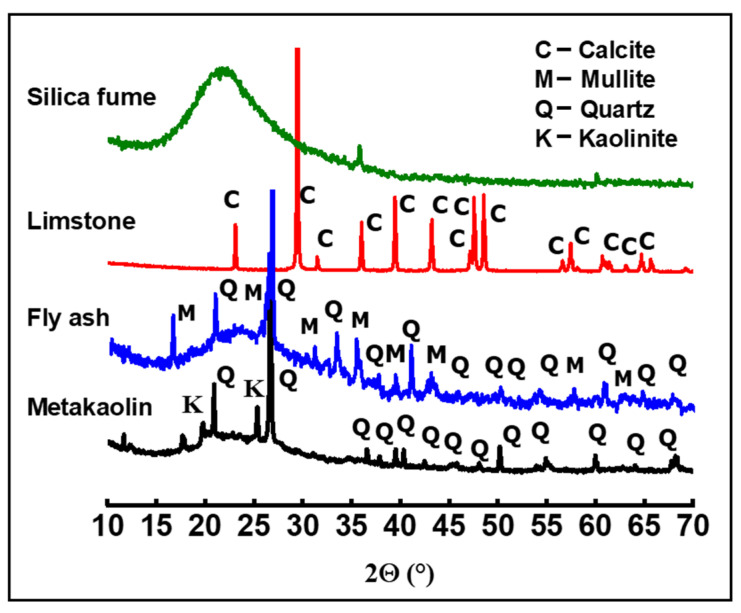
XRD patterns of metakaolin, fly ash, limestone, and silica fume powders.

The nature of the crystalline phase present in the powders was investigated by using X-ray diffraction measurements. XRD patterns were obtained at room temperature using a Bruker D8 (Bruker, Madison, WI, USA), employing CuKα radiation as the X-ray source (operating voltage was 40kV and current was 40 mA). XRD data were collected in the 10–70° 2θ range, with a 0.059° step-scan and at 5 s per step. The XRD patterns of metakaolin (MK), fly ash (FA), limestone (LM), and silica fume (SF) are given in Figure 1. All XRD peaks of limestone can be attributed to calcite (JCPDS: 88-1807). XRD reveals the amorphous nature of the silica fume. The XRD pattern of metakaolin shows a mixture of quartz (JCPDS: 46-1045) and residual kaolinite (JCPDS: 29-1488) in addition to the main amorphous phase. The fly ash powder is largely amorphous, with a small amount of mullite (JCPDS:15-0776) and quartz phases. These results are in perfect agreement with the XRF analysis.

**Figure 2:**
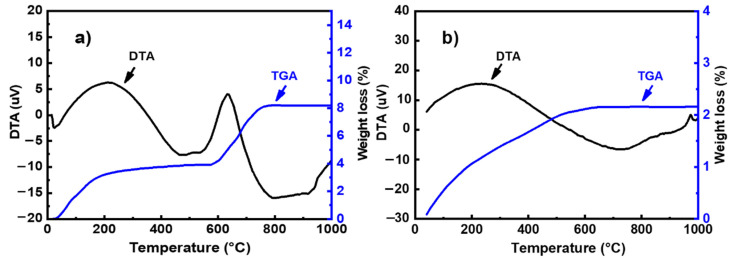
TG–DTA curves for (**a**) metakaolin and (**b**) fly ash.

**Figure 3:**
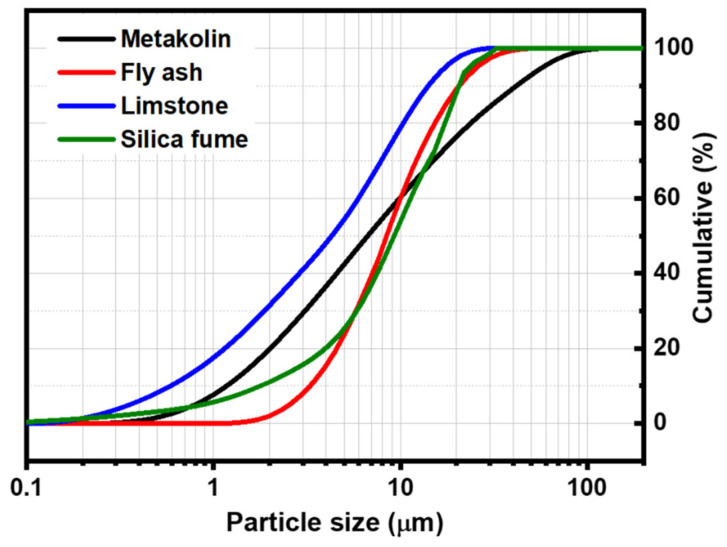
Particle size distribution of input material powders.

The thermal behavior of powders was studied using a DTG 60 instrument from Shimadzu (Shimadzu, Kyoto, Japan). Analyses were performed in air using a heating rate of 10 °C/min. The DTA-TG curves of metakaolin and fly ash is presented in Figure 2. The thermal decomposition of fly ash occurred in two steps (Figure 2a): In the first step, the mass loss in the TG curve below 100 °C can be attributed to the departure of adsorbed water. This phenomenon is associated with endothermic effects at 20–75 °C in the DTA curve. In the second step, a mass loss of about 8% is observed in the temperature range of 600–800 °C in the TG graph, accompanied by an exothermic peak in the DTA curve. This weight loss could be attributed to the decomposition of calcite (CaCO_3_) present in the sample [12]. The TG curve of metakaolin (Figure 2b) indicates that there are several mass losses up to 650 °C. These could be attributed to the evaporation of adsorbed water and the dehydroxylation of residual kaolinite (Al_2_Si_2_O_5_(OH)_4_). The weak exothermic peak observed at around 994 °C in the DTA curve can be related to the crystallization of mullite [13,14]. The particle size distribution was investigated with a Laser analyzer Mastersizer 2000 instrument (Malvern Instruments, Worcestershire, UK). Figure 3 shows the particle size distribution curves of all raw materials. These curves show a d_50_ of 6.67, 8.44, 4.27, and 9.31 μm for metakaolin, fly ash, limestone, and silica fume, respectively.

### 2.2. Preparation of Geopolymer-Based Materials

**Figure 4:**
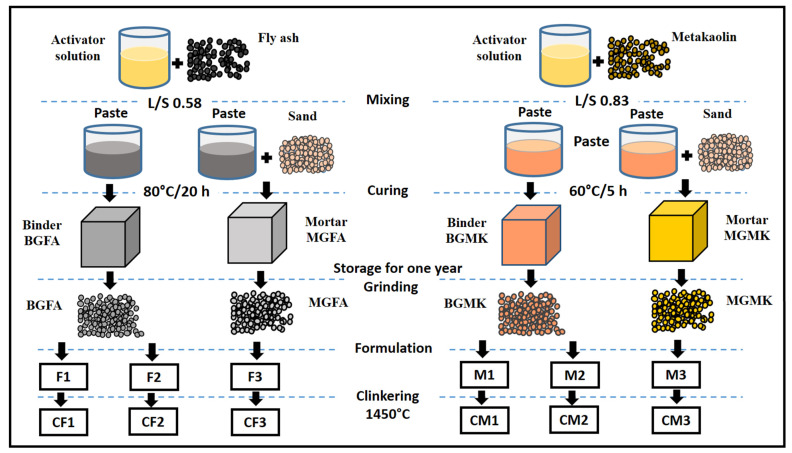
Flowchart representing the procedure for the preparation of cement clinker from geopolymer-basedmaterials.

The preparation of geopolymer materials was carried out in accordance with our previous study [8]. The procedure for geopolymer preparation is shown in Figure 4. The geopolymer materials were obtained through thereaction between an activator solution and the metakaolin or fly ash powders. Initially, the activator solution (AS), with a weight ratio of SiO_2_/Na_2_O = 1.2, was prepared by mixing sodium silicate, sodium hydroxide, and water. The obtained solution was stirred for 24 h at room temperature before use. Then, the activator solution was mixed with the metakaolin or fly ash powders. After that, the mixture was mechanically mixed until the homogenization of the geopolymer pastes. The weight ratio of the liquid (AS)/aluminosilicate source (metakaolin or fly ash) was 0.83 and 0.58 for the metakaolin-based geopolymer binder (BGMK) and the fly ash-based geopolymer binder (BGFA), respectively. To prepare geopolymer mortars, standard sand was added to the geopolymer paste atthe weight ratio of sand:aluminosilicate source = 70:30. The obtained materials were named MGMK and MGFA for metakaolin and fly ash-based mortars, respectively. The geopolymer binders and mortars were cast into polypropylene molds and vibrated to remove air bubbles. Then, the molds were sealed with a polyethylene film to prevent the rapid evaporation of water. The specimens were then cured at 60 °C/5 h and 80 °C/20 h for metakaolin-based materials (BGMK and MGMK) and fly ash-based materials (BGFA and MGFA), respectively. The geopolymer materials were stored for a year in open air in order to simulate aging in an ambient environment andwere subsequentlycrushed using a roller mill. The geopolymer materials were ground into fine powders to be able to use them as raw materials that would replace clay in theproduction of cement clinkers by the conventional sintering process.

### 2.3. Clinker Preparation

It is well-known that the chemical composition of a geopolymer binder/mortar is very different from that of a cement clinker [15]. Therefore, geopolymer powders are mixed with calcium carbonate and corrective materials to adjust the clinker formulation. The preparation of cementclinker is controlled by the lime saturation factor (LSF), silica ratio (SR), and alumina ratio (AR), which are calculated according to Equations (1)–(3):

LSF = (C/(2.8S + 1.2A + 0.6F))(1)

SR = (S/(A + F))(2)

AR = (A/F)(3)

where C, S, A, F are CaO, SiO_2_, Al_2_O_3_, and Fe_2_O_3_, respectively.

The values of LSF, SR, and AR were setat 92–98, 2–3.7, and 1.0–4.0, respectively (ASTM C 150-97). As shown in Table 2, six formulations were investigated in this study. The ingredients of formulations were calculated according to the chemical composition of raw materials (Table 1 and Table 3). To investigate the reactivity of geopolymer amorphous phases, we designed formulations F1 and M1 by replacing a high proportion of mineral clay (>67 wt%) with geopolymer BGMK and BGFA binders. The formulations F2, M2, F3, and M3 were designed to produce clinker with low Na_2_O content. The reaction mixtures were ground to ensure good homogeneity. Then, they were poured into a platinum crucible and heated in a muffle furnace under air at a temperature rate of 5 °C/min. The firing cycle involved three steps: (i) heating at 1000 °C for 1 h in order to promote the complete decomposition of carbonates, (ii) heating at 1450 °C for 1 h to promote the complete crystallization of the calcium-silicate and calcium-iron-aluminate phases, (iii) air quenching to prevent the crystallization of the γ-C_2_S phase.

## 3. Results and Discussion

### 3.1. Geopolymer-Based Raw Materials

**Figure 5:**
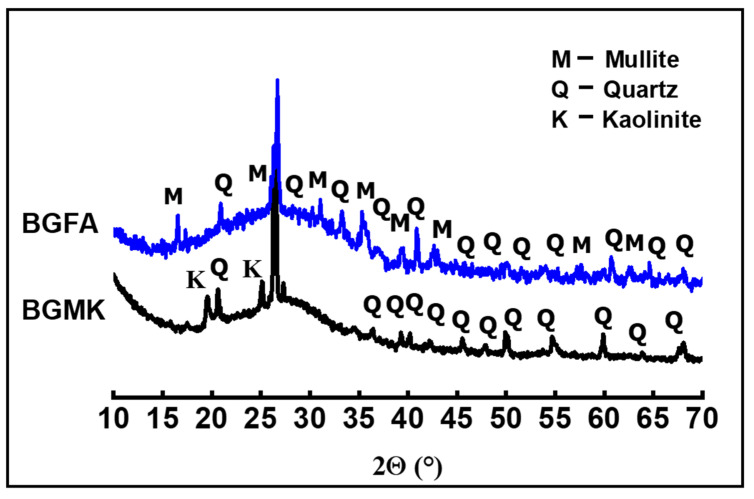
XRD patterns of BGMK and BGFA geopolymer binders.

**Figure 6:**
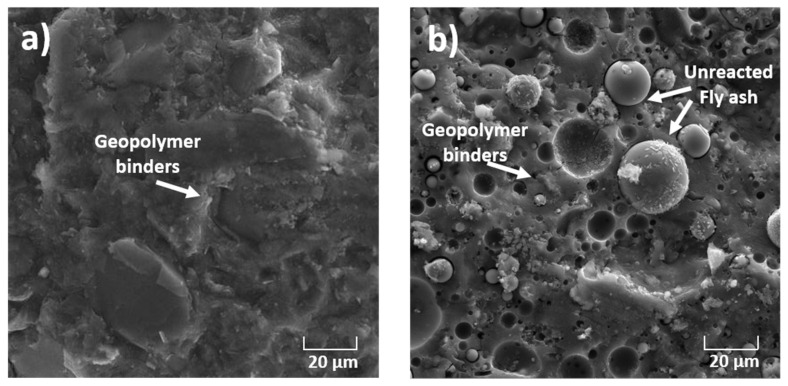
SEM micrographs of (**a**) BGMK and (**b**) BGFA geopolymer binders.

As mentionedabove, one of the main objectives of this work was to demonstrate the reactivity and the conversion of geopolymer amorphous phases into clinker phases. Simulated geopolymer-based material wastes were prepared using two aluminosilicate sources: metakaolin and fly ash. The aluminosilicate source was activated by an alkaline silicate solution. This solution provided sufficient alkalinity to dissolve the amorphous aluminosilicate powders while inhibiting the formation of a gel at an early stage, thus covering the aluminosilicate particles, which hindered further dissolution. Moreover, the activating solution providedthe soluble silicate species necessary for the polycondensation reaction to obtain a compact connected network with a high degree of geopolymerization [16,17,18,19]. The XRD patterns of geopolymer binders are given in Figure 5. The XRD pattern of the metakaolin-based geopolymer shows peaks characteristic of quartz and residual kaolin. These crystalline phases were initially present in the metakaolin precursor as impurities and resisted in the activation process. However, the XRD pattern of fly ash-basedgeopolymer shows a mixture of quartz and mullite phases. These refractory phases resisted the activation process. Both patterns show a broad halo peak at a low 2-theta position (between 20° and 40°), which indicates the presence of amorphous phases. These results agree well with those recently reported in the literature [18,19]. It is well-known that the geopolymer phases are amorphous in nature. In our case, all the observed XRD peaks can be attributed to the unreacted crystal precursors. The amorphous phases observed in our materials represent mainly the formation of sodium aluminosilicate gel. The samples were also investigated with a scanning electron microscope (Tescan VEGA 3) (Tescan Orsay Holding, Brno-Kohoutovice, Czech Republic) provided with energy-dispersive X-ray spectroscopy (EDS) (Oxford Instruments, Oxford, UK). As seen in the SEM micrographs (Figure 6), the two geopolymer binders are composed mainly of amorphous phases, indicating a high degree of geopolymerization [20,21]. Close inspection of the SEM images revealed the presence of the unreacted precursor particles in the fly ash-basedgeopolymer (Figure 6b) [21,22].

**Figure 7:**
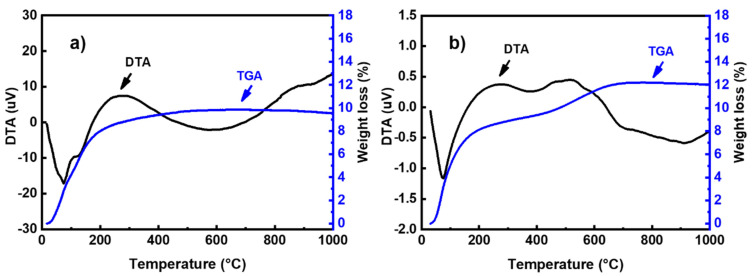
TGA/DTA curves of (**a**) BGMK and (**b**) BGFA geopolymer binders.

The thermal behavior of geopolymer binders was investigated by thermal analysis (Figure 7). As seenin Figure 7a, the TG curve of the BGMK binder shows a successive mass loss of about 8% below 200 °C. These weight losses are accompanied by two endothermic peaks at 75 °C and 125 °C in the DTA curve. According to Rodríguez et al. [23], these mass losses originated inthe free water trapped in the pores and zeolitic water available in the reaction products, which can easily be removed from the alkaline silicategel surface at this temperature. However, the TGA curve of the fly ash-based geopolymer (Figure 7b) shows weight loss in two steps. The weight loss observed below 200 °C, associated with an endothermic peak at 75 °C in the DTA curve, can be attributed to the departure of adsorbed water from the sample [24]. A second mass loss of about 4% is observed in the temperature range of 450–650 °C, accompanied by exothermic effects in the DTA curve. This weight loss could be assigned to the decomposition of calcium hydroxide (Ca(OH)_2_) [21,25].

**Figure 8:**
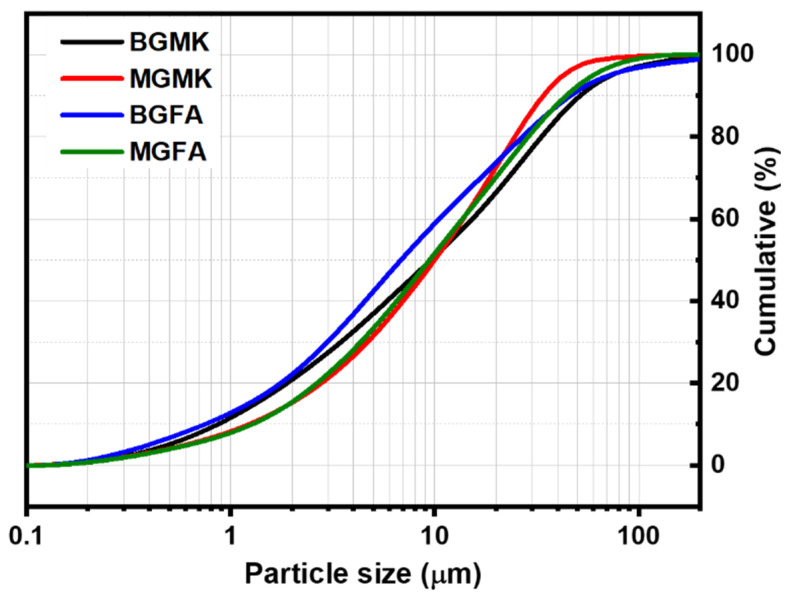
Particle size distribution curves for the ball-milled geopolymer wastes.

The chemical composition of geopolymer binders and geopolymer mortars are given in Table 3. The geopolymer binders are mainly composed of SiO_2_ and Al_2_O_3_. The relatively high content of Na_2_O (>6%) in both geopolymer binders comes from the activator solution. The chemical composition of the geopolymer mortars MGFA and MGMK are similar, with minor differences in their oxide content. Their SiO_2_ content is higher than 80%, which is due to the presence of standard sand. However, the Al_2_O_3_ content of the geopolymer mortars is relatively low compared to geopolymer binders. The geopolymer-based materials are composed mainly of SiO_2_ and Al_2_O_3_, which are suitable substitutes of mineral clays in the preparation of cement clinker [26]. Moreover, these materials contain low amountsof CaO, Fe_2_O_3_, and MgO. In order to use the geopolymer-based materials as raw materials instead of clay in the preparation of cement clinker, they were first crushed to reduce their initial size. Then, they were loaded into the ball mill for the final grinding. It has beendemonstrated by many research groups that particle size plays a crucial role in the solid-state reaction [27]. Figure 8 shows the particle size distribution of the different geopolymer-based powders. As can be seen, all geopolymer-based materials were reduced to powders composed of fine particles (d_50_ between 7 and 10 μm).

### 3.2. Cement Clinkers

Several steps have been proposed to explain the formation of cement clinker [28]:

(i) Formation of intermediate reactive oxides by dehydration/dehydroxylation and decomposition of clay at temperatures lower than 950 °C;
(ii) Formation of metal oxides (i.e., CaO, MgO) by carbonate decomposition at 550–1000 °C;
(iii) Crystallization of the calcium aluminate CA (CaO·Al2O3) and ferroaluminate C2AF (2CaO·Al2O3·Fe2O3). Then, conversion of CaO, CS, CA, and Fe2O3 into the ferrite C4AF, tricalcium aluminate C3A, and belite C2S phases. These reactions occur in the temperature range of 550–1280 °C, according to the following Equations:
  CaO·Al_2_O_3_ + 2CaO → 3CaO·Al_2_O_3_ (4)
  CaO·Al_2_O_3_ + 3CaO + Fe_2_O_3_→ 4CaO·Al_2_O_3_·Fe_2_O_3_ (5)
  CaO·SiO_2_ + CaO → 2CaO·SiO_2_ (6)
(iv) Formation of the alite phase C_3_S by the chemical reaction of free CaO with C_2_S; melting of C_4_AF attemperatures higher than 1280 °C. The molten C_4_AF medium promotes the growth of the pre-existing C_2_S and C_3_S phases in the reaction mixture at this temperature range.

The chemical composition of the investigated clinkers are given in Table 4. As can be seen, the chemical composition of all clinkers is very similar, except the Na_2_O content. The high alkali content observed in the CM1 and CF1 clinkers was caused by the high amount of geopolymer binders incorporated into the reaction mixture (Table 2). As discussed previously, the Na_2_O content of geopolymer binders is greater than 6 wt% (Table 3). The alkali content of cement clinkers is a crucial parameter that influences the properties of concrete. Indeed, the high alkali content of the clinker induces the reaction of Na_2_O/K_2_O with aggregates in concrete, thus causing the expansion and cracking of the materials [29]. The alkali equivalent content Na_2_Oeq was calculated according to Equation (7) [30].

Na_2_Oeq = Na_2_O + 0.658 K_2_O (7)

As seen in Table 5, the alkali equivalent content (Na_2_Oeq) values of the formulations CM1 and CF1 are larger than the maximum acceptable value defined by the Standard ASTM C 150-97 [30]. However, when the natural clay mineral was replaced withan adequate proportion of geopolymer-based material (clinkers CM2, CF2, CM3, and CF3), the alkali equivalent content (Na_2_Oeq) was less than 0.6 wt%.

The mineralogical composition of the investigated clinkers determined according to Bogue equations (Equations (8)–(11)) is shown in Table 5. The C_3_S, C_2_S, C_3_A, C_4_AF phase contents of all clinkers are very similar and conform to the standard specification for Portland cement clinker. Furthermore, the lime saturation factor (LSF), silica ratio (SR), and alumina ratio (AR) of all cement clinkers are in the recommended ranges for Portland cement (Table 5). These results indicate the ideal amountof lime, silica, alumina, and iron oxide in our formulations. Free lime (f-CaO) content detected by the glycerol-ethanol method is less than 1.3 wt% for all clinkers, which indicates a relatively high degree of clinkerization [31,32,33].

C_3_S (%) = 4.071(CaO) − 7.602(SiO_2_) − 6.718(Al_2_O_3_) − 1.43(Fe_2_O_3_) (8)

C_2_S (%) = 2.87(SiO_2_) − 0.754(C_3_S) (9)

C_3_A (%) = 2.65(Al_2_O_3_) − 1.692(Fe_2_O_3_) (10)

C_4_AF (%) = 3.043(Fe_2_O_3_) (11)

**Figure 9:**
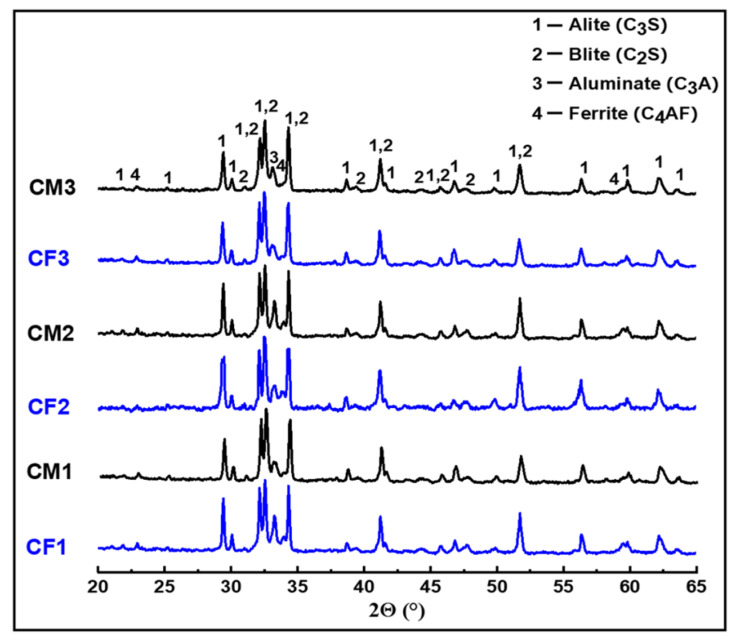
XRD patterns of the prepared clinkers.

The XRD patterns of the prepared clinkers are presented in Figure 9. In the patterns of all clinkers fired at 1450 °C, mixtures of four phases were identified: alite C_3_S (JCPDS: 49-0442), belite C_2_S (JCPDS: 33-0302), tricalcium aluminate C_3_A (JCPDS: 38-1429), and ferrite C_4_AF (JCPDS: 30-0226). The characteristic XRD peaks of lime were not observed in any pattern, which indicates a very low CaO_f_ content in our clinkers. Likewise, the γ-C_2_S phase was not crystallized in any of our samples, which is probably due to the quenching treatment [34]. The high degree of crystallization of clinker phases and the low amountof free lime suggests a high degree of clinkerization.

**Figure 10:**
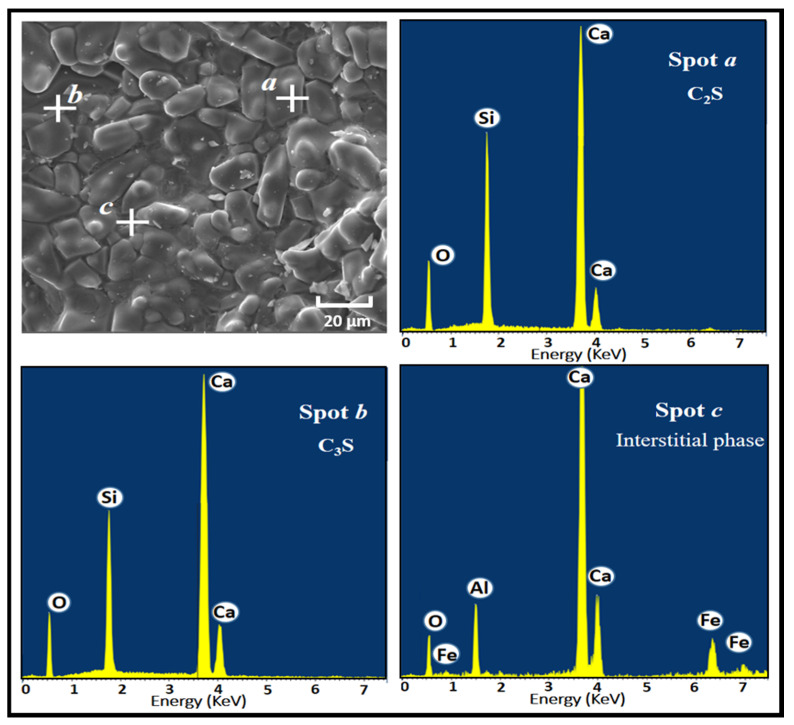
SEM micrograph and EDS spectra of the cement clinker CF3.

Furthermore, the microstructure of the prepared clinkers was investigated by SEM-EDS analysis. The SEM image of clinker CF3 along with the energy spectra is represented in Figure 10 as a typical example. The C_3_S crystal is seen to have a different form and a size less than 40 µm. Small C_2_S grains are also observed in the SEM image. Finally, the interstitial phase observed in the SEM image is composed of Ca, Si, Al, and Fe, which can be attributed to C_4_AF and C_3_A melting together and interweaving with each other.

## 4. Conclusions

In this study, cement clinkers were prepared using geopolymer-based materials as raw materials. Four different geopolymer-based materials were investigated: the metakaolin-based geopolymer binder (BGMK) and mortar (MGMK), and the fly ash-based geopolymer binder (BGFA) and mortar (MGFA). The chemical analysis carried out by XRF revealed that these materials are very rich in silica and alumina, which can be suitable substitutes of mineral clays in the preparation of clinker. In all investigated formulations, the crystalline phases identified by XRD after clinkerization at 1450 °C were C_3_S, C_2_S, C_4_A, C_4_AF. The free lime content was less than 1.3 wt% in all samples, indicating a high degree of clinkerization. Chemical analysis revealed that the amount of geopolymer wastes incorporated into the clinker mixture was limited by the equivalent alkali (Na_2_Oeq) content. The Na_2_Oeq content was kept at less than 0.6 wt% when the natural clay was substituted with 17.3 wt%, 11.8 wt%, 61.8 wt%, and 43.50 wt% of BGMK, BGFA, MGMK, and MGFA, respectively. We expect these results toprovide a theoretical basis for the recovery of geopolymer wastes in the production of cement clinker.

## Figures and Tables

**Table 1:** Chemical composition of metakaolin, fly ash, limestone, silica fume, and sand.

Raw Materials	Oxides (wt%)									
	SiO_2_	Al_2_O_3_	Fe_2_O_3_	CaO	MgO	SO_3_	Na_2_O	K_2_O	P_2_O_5_	LOI
**Metakaolin**	57.38	37.11	1.41	0.43	0.36	0.19	0.18	0.81	0.06	2.08
**Fly Ash**	51.48	22.57	5.86	4.79	2.21	0.51	0.62	1.10	0.86	10.01
**Limestone**	0.21	0.08	0.02	56.13	0.26	0.20	0.01	0.01	0.01	43.08
**Silica Fume**	93.86	0.27	0.04	0.96	1.28	0.20	0.18	0.30	0.09	2.82
**Sand**	97.81	0.45	0.06	0.39	0.08	0.22	0.10	0.24	0.02	0.63

**Table 2:** Investigated formulations (wt%).

Raw Materials	Formulations (wt%)					
	M1	F1	M2	F2	M3	F3
**Limestone**	76.86	74.01	78.35	76.83	78.63	76.93
**Metakaolin**	-	-	6.76	-	6.50	-
**Fly Ash**	-	-	-	12.17	-	12.19
**BGMK**	15.53	-	3.74	-	-	-
**BGFA**	-	21.08	-	2.75	-	-
**MGMK**	-	-	-	-	13.20	
**MGFA**	-	-	-	-		10.04
**Silica fume**	5.82	4.30	9.49	7.41	-	-
**Fe_2_O_3_**	1.79	0.60	1.66	0.85	1.67	0.85

**Table 3:** Chemical composition of geopolymer-based materials.

Raw Materials	Oxides (wt%)									
	SiO_2_	Al_2_O_3_	Fe_2_O_3_	CaO	MgO	SO_3_	Na_2_O	K_2_O	P_2_O_5_	LOI
**BGFA**	47.05	17.34	5.49	5.45	2.12	0.55	6.64	1.66	0.58	13.11
**BGMK**	54.42	25.35	1.09	1.21	0.28	0.31	8.06	0.68	0.10	8.49
**MGFA**	84.02	5.04	1.53	1.77	0.64	0.31	1.88	0.63	0.17	4.02
**MGMK**	84.79	7.92	0.37	0.64	0.14	0.25	2.49	0.37	0.04	2.99

**Table 4:** Chemical composition of the produced cement clinkers.

Portland Cement Clinker	Oxides (wt%)									
	SiO_2_	Al_2_O_3_	Fe_2_O_3_	CaO	MgO	SO_3_	Na_2_O	K_2_O	P_2_O_5_	LOI
**CM1**	21.42	6.10	2.97	65.95	0.49	0.32	1.88	0.19	0.04	0.64
**CF1**	21.48	5.66	2.64	65.25	1.06	0.42	2.15	0.57	0.20	0.57
**CM2**	22.76	5.38	2.72	66.98	0.55	0.30	0.48	0.16	0.03	0.63
**CF2**	22.41	5.19	2.66	67.00	0.95	0.37	0.38	0.30	0.20	0.52
**CM3**	22.86	5.32	2.74	67.13	0.38	0.31	0.48	0.16	0.02	0.62
**CF3**	22.70	5.04	2.58	67.05	0.81	0.38	0.38	0.30	0.19	0.58

**Table 5:** Mineralogical composition of the prepared cement clinkers.

Mineralogical Phases (%)	Portland Cement Clinker						ASTM C 150-97
	CM1	CF1	CM2	CF2	CM3	CF3	
**C_3_S**	55.79	55.47	54.75	58.75	54.8	57.70	50–70
**C_2_S**	19.65	20.05	24.27	20.25	24.5	21.88	15–35
**C_3_A**	11.15	10.53	9.66	9.24	9.46	8.99	5–12
**C_4_AF**	9.02	8.04	8.27	8.09	8.32	7.84	5–15
**Quality Indexes**							
**SR (%)**	2.36	2.59	2.81	2.86	2.84	2.98	2.0–3.7
**AR (%)**	2.06	2.14	1.98	1.95	1.94	1.95	1.0–4.0
**LSF (%)**	94	93	92	93	92	92	92–98
**Na_2_Oeq (wt %))**	2.01	2.52	0.59	0.58	0.59	0.58	0.6
**CaO_f_ (wt %))**	1.12	1.23	1.18	1.23	1.25	1.26	0.5–1.5
